# Outcomes of Surgical Pulmonary Embolectomy for Acute Pulmonary Embolism: A 14-Year Single-Centre Study

**DOI:** 10.7759/cureus.101721

**Published:** 2026-01-17

**Authors:** Nada Ali, Ibrahim Warda, Agni L Salem, Amer Harky, Mohamed Zeinah

**Affiliations:** 1 Cardiothoracic Surgery, Liverpool Heart and Chest Hospital NHS Foundation Trust, Liverpool, GBR; 2 General Practice, Imperial College Healthcare NHS Trust, London, GBR

**Keywords:** acute pulmonary embolism, cardiothoracic surgery, high-risk pulmonary embolism, retrospective cohort study, surgical pulmonary embolectomy

## Abstract

Objective: This retrospective single-centre cohort study investigates the outcomes of surgical pulmonary embolectomy for high-risk acute pulmonary embolism (PE) at our institution over a 14-year period.

Methods: We conducted a retrospective review of medical records for 13 consecutive patients who underwent surgical pulmonary embolectomy (SPE) between 2006 and 2020. Data collected included patient demographics, comorbidities, pre-operative assessments (echocardiography, computed tomography pulmonary angiography (CTPA) findings), indications for SPE, surgical details, post-operative complications, and 12-month survival.

Results: The study cohort comprised 13 patients, predominantly male (69.2%), with a median age of 47 years. Comorbidities varied, with one patient lacking identifiable risk factors. Pre-operative assessment demonstrated right ventricular dysfunction identified on echocardiography, CTPA, or both modalities. Indications for SPE included high-risk PE (76.9%), contraindication to thrombolysis (7.7%), and persistent haemodynamic instability despite thrombolysis (15.4%). All procedures were performed via median sternotomy with cardiopulmonary bypass. The median ICU stay was two days. In-hospital mortality was 23.1%. Post-operative complications included atrial fibrillation (30.8%), cardiac tamponade (15.4%), and residual PE (23.1%). Twelve-month survival was 76.9%.

Conclusion: Our 14-year experience demonstrates that SPE remains a viable and critical intervention for select high-risk PE patients, particularly in a specialised tertiary care setting. Despite the critical nature of the patient population, the observed outcomes align with contemporary reports, suggesting improved survival with timely and expert care.

## Introduction

Surgical pulmonary embolectomy (SPE) was first described in the early 20th century and historically carried high mortality, limiting its use to rescue situations. Outcomes improved substantially after the introduction of cardiopulmonary bypass and the refinement of modern perioperative care [1-4].

Pulmonary embolism (PE), a major manifestation of venous thromboembolism (VTE), remains a leading cause of cardiovascular mortality, particularly in patients who present with haemodynamic instability [5,6]. Current guidelines recommend SPE for high-risk PE in unstable patients who have contraindications to thrombolysis or who fail systemic or catheter-directed therapy [6-8].

An emerging literature body now questions whether SPE should be considered earlier, before clinical deterioration, given increasingly favourable outcomes in specialised centres [9-12].

Our study primarily aimed to descriptively evaluate 12-month survival following SPE in a high-risk cohort. Secondary objectives were to characterise the indications for surgical embolectomy and the pre-operative clinical and imaging features of patients undergoing this intervention. This study was exploratory and descriptive in nature, and no comparative or inferential analyses were intended.

We therefore undertook a 14-year retrospective review of SPE at our tertiary centre, adding to existing literature and contributing to ongoing discussion regarding the role and timing of surgical intervention in acute PE.

## Materials and methods

Study design and population

This study was conducted as a retrospective, observational, single-centre study at a tertiary cardiothoracic centre. All adult patients who underwent SPE between January 2006 and December 2020 were reviewed.

Patients were identified consecutively using institutional coding systems and paper-based archives, depending on the time period. Inclusion criteria comprised all adult patients who underwent SPE during the study period. Of the identified patients, one was excluded due to incomplete clinical records that precluded meaningful analysis, and two were excluded because PE occurred as an intraoperative complication of another surgical procedure at the same institution. The final study cohort consisted of 13 patients.

Data collection

Clinical data were collected retrospectively from electronic medical records or archived paper case notes, depending on the time of surgery. Extracted variables included patient demographics, comorbidities and risk factors, pre-operative imaging findings, indications for surgical intervention, operative and procedural details, and post-operative outcomes. Data were extracted using a predefined data collection proforma and independently verified by two authors, with all data recorded in a de-identified format.

Follow-up duration was capped at 12 months in accordance with the predefined survival outcome, with survival status determined from hospital records and follow-up documentation, and deaths verified using institutional clinical records.

With respect to missing data, pre-operative echocardiographic findings were unavailable for one patient; all other variables were complete. No data imputation was performed, and analyses were conducted using available-case data only.

Statistical analysis

Given the small cohort size and low event rates, analyses were limited to descriptive statistics. Continuous variables are presented as medians with ranges, and categorical variables as counts and percentages. No comparative or inferential statistical analyses were performed, and multivariable regression modelling was not attempted, as such analyses would be underpowered and unreliable in this cohort. Statistical tabulation and analyses were conducted using Microsoft Excel (Microsoft Corporation, Redmond, WA).

Governance and ethical considerations

The study was conducted as a service-evaluation audit under local institutional governance frameworks using routinely collected, de-identified patient data. 

## Results

A total of 13 patients underwent SPE between 2006 and 2020. Key demographic, clinical, and perioperative details are summarised in Table 1. The cohort was predominantly male (n=9, 69.2%), with a median age of 47 years (range: 20-79 years). Comorbidities and risk factors varied widely and included immobility, obesity, metastatic malignancy, protein C deficiency, and pancreatitis. One patient presented without identifiable risk factors.

**Table 1:** Patient Characteristics, Imaging Findings, Procedural Details, and 12-Month Survival Following Surgical Pulmonary Embolectomy This table summarizes baseline demographics, comorbidities, and risk factors, pre-operative echocardiographic findings, computed tomography pulmonary angiography (CTPA) results, indications for surgical intervention, operative and procedural details, postoperative complications, and 12-month survival for patients undergoing surgical pulmonary embolectomy (SPE). High-risk pulmonary embolism was defined by the presence of hemodynamic instability, including sustained hypotension, cardiogenic shock, or the need for cardiopulmonary resuscitation, in accordance with contemporary guideline criteria [6]. Abbreviations: AF, atrial fibrillation; CAD, coronary artery disease; CPB, cardiopulmonary bypass; CTPA, computed tomography pulmonary angiography; CVP, central venous pressure; DVT, deep vein thrombosis; ECHO, echocardiography; HAP, hospital-acquired pneumonia; HTN, hypertension; IBD, inflammatory bowel disease; ICU, intensive care unit; IVC, inferior vena cava; LA, left atrium; LV, left ventricle; MOF, multiorgan failure; PA, pulmonary artery; PE, pulmonary embolism; PFO, patent foramen ovale; RA, right atrium; RV, right ventricle; RVSP, right ventricular systolic pressure; TR, tricuspid regurgitation; Y, yes; N, no

Patient	Age/Sex	Comorbidities & Risk factors	Pre-operative ECHO	CTPA	Indications for surgical intervention	Procedure details	Caval filter	CPB time (min)	ICU stay (days)	Post-operative Complications	12-month survival
1	79 M	MI; PA Sarcoma; IBD	Hypertrophic RV, raised RVSP, mass in the main PA	Massive filling defect in the pulmonary trunk extending to the left PA	High-risk PE	Pulmonary embolectomy and pulmonary trunk mass excision	No	145	6	AF	Y
2	28 M	Protein C deficiency	Dilated RV & impaired function	Saddle PE	High-risk PE	Saddle embolectomy	Yes	21	2	Pericardial effusion	Y
3	56 F	DVT; HTN; liver cirrhosis; hospital admission for oesophageal varices	Not available	Saddle embolus in the pulmonary trunk extending into both PA	Thrombolysis contraindicated	Saddle embolectomy	Yes	25	3	None	Y
4	32 F	DVT; poor mobility due to schizophrenia and catatonia	RV not particularly dilated; moderately raised CVP	Saddle embolus in the pulmonary trunk extending into both PA	High-risk PE	Saddle embolectomy	No	55	3	Residual PE; fulminant liver failure	N
5	67 M	Hospital admission for wrist fracture	Severely dilated RV with poor function	Massive left pulmonary embolus causing complete occlusion; emboli in right distal PA; RV strain	High-risk PE	Left main PA completely occluded with thrombus; embolectomy from the right PA	No	76	2	Acute hepatic necrosis	N
6	47 M	Bed-bound secondary to scalded skin syndrome	Reduced RV function and right heart strain	Saddle embolus at the pulmonary trunk; multiple extensive pulmonary emboli seen throughout both sides of the pulmonary arterial tree; right heart strain; small segment of pulmonary infarction	Unstable despite trial of thrombolysis	Bilateral pulmonary embolectomy	Yes	40	1	Pericardial effusion and cardiac arrest necessitating surgical drainage	Y
7	64 M	HTN; CAD	LV mildly impaired, RV mildly impaired	Large bilateral PE	High-risk PE; right atrial embolus straddling the PFO	Bilateral pulmonary embolectomy; closure of PFO	No	74	2	AF; HAP	Y
8	38 M	Hospital admission for wrist fracture	RV dilated with impaired systolic function, hyperkinetic LV	Large PE in the proximal left PA	High-risk PE	Embolectomy left PA	Yes	21	2	Residual PE	Y
9	21 M	Obesity	Dilated impaired RV	Extensive bilateral PE; large filling defect in IVC	High-risk PE	Bilateral pulmonary embolectomy	No	9	7	Residual PE; tamponade and cardiac arrest requiring re-sternotomy	Y
10	64 M	Recent long-haul flight	Dilated RA & LA with bilateral PA thrombus	Big clot within right & left PA; large RA thrombus extending to LA through PFO	High-risk PE	Bilateral pulmonary embolectomy; removal of mass in RA & LA with closure of PFO	No	212	6	Severe biventricular failure and MOF	N
11	60 F	Pancreatitis	Dilated RA with large clot in RA passing through tricuspid valve	Saddle pulmonary embolus extending to the distal main pulmonary arteries bilaterally	High-risk PE; saddle PE extending into RA and RV	Bilateral pulmonary embolectomy	No	23	2	AF	Y
12	30 M	Metastatic testicular tumor	Large thrombus in the RA, preserved LV and RV function	Multiple bilateral PE in both pulmonary arteries	Unstable despite trial of thrombolysis; right atrial myxoma	Pulmonary embolectomy; RA atrial mass extraction	Yes	34	1	AF; cardiac tamponade requiring surgical drainage	Y
13	20 F	No identifiable risk factor	Distended RV, with poor function; distended RA; severe TR	Massive bilateral pulmonary emboli; right heart strain	High-risk PE	Bilateral pulmonary embolectomy	No	55	4	None	Y

Pre-operative imaging demonstrated right ventricular dysfunction on echocardiography or computed tomography. Furthermore, investigations revealed that one patient had a patent foramen ovale (PFO), another had PFO with thrombus in the right atrium (RA) and left atrium (LA), and a third patient had a large thrombus in the RA.

High-risk PE was the indication in 10 patients (76.9%), with an additional two presenting with haemodynamic instability despite thrombolysis (15.4%) and one with contraindications to thrombolytic therapy (7.7%). All operations were performed via median sternotomy with cardiopulmonary bypass. The median bypass time was 40 minutes (range: 9-212 minutes). Inferior vena cava filters were inserted in five patients (38.5%).

Post-operatively, the median intensive care stay was two days (range: 1-7 days). The in-hospital mortality was 23.1% (n=3). Causes of death included acute liver failure (n=2, 66.7%) and biventricular failure, resulting in multiorgan failure (n=1, 33.3%). Atrial fibrillation was the most frequent complication (n=4, 30.8%), followed by residual PE (n=3, 23.1%) and cardiac tamponade requiring surgical drainage (n=2, 15.4%). Other complications included pericardial effusion, hepatic failure, biventricular failure, multiorgan failure, and hospital-acquired pneumonia. The 12-month survival rate for the cohort was 76.9% (n=10).

## Discussion

This 14-year retrospective review offers insight into contemporary SPE practice within a specialised tertiary centre. Our patient cohort reflects the wide clinical spectrum of acute PE, where symptoms range from dyspnoea and chest pain to syncope - often a marker of haemodynamic instability or significant right ventricular (RV) dysfunction [6,13]. RV dysfunction, demonstrable on echocardiography or computed tomography, is a key determinant of short-term prognosis [14]. Consistent with known VTE epidemiology, our cohort exhibited multiple established risk factors such as immobility, obesity, malignancy, and thrombophilia, although a substantial proportion of VTE cases still arise without identifiable triggers [15-17].

Current management of PE is based on a risk stratification approach, with high-risk PE defined as PE associated with haemodynamic instability, including cardiac arrest, obstructive shock, or sustained hypotension despite adequate volume status, in the absence of alternative causes [6].

Historically, SPE was considered a heroic, last-resort intervention, with early mortality rates approaching 50% [18]. Its development in the early 20th century by Trendelenburg and Kirschner was hampered by the absence of cardiopulmonary bypass (CPB), and meaningful improvements only occurred after CPB became available, including its first successful application to embolectomy by Sharp in 1962 [1-4]. Subsequent refinements in perioperative care and surgical technique have contributed to markedly improved outcomes over time.

Our in-hospital mortality of 23.1% is consistent with contemporary series reporting rates of 22-25% [9,10,12,19,20]. These outcomes reflect the high acuity of the patients typically referred for SPE - often those who have failed thrombolysis or present with severe haemodynamic compromise.

Current guideline recommendations position SPE as a treatment for high-risk PE in patients who are unstable, have contraindications to thrombolysis, or fail systemic or catheter-directed reperfusion therapies [6,8]. Additional indications include embolus-in-transit within the right heart or across a patent foramen ovale [21-23]. The risks associated with thrombolysis, particularly major bleeding and intracranial haemorrhage, emphasise the value of surgical intervention in appropriately selected patients [24-26].

A growing debate centres on whether SPE should be considered earlier in the treatment pathway. Emerging evidence suggests that delayed referral, often after repeated thrombolysis attempts or prolonged haemodynamic instability, may contribute more to poor outcomes than the procedure itself [27-29]. It remains unclear whether the persistently poor outcomes ascribed to SPE stem from its intrinsic risk or from the late referral of patients already in extremis. Reports from specialised centres support the notion that early multidisciplinary evaluation, rapid surgical access, and timely intervention may improve survival [9-12,30]. Our own experience, though limited by a small sample size, aligns with this evolving understanding, suggesting that the timing of referral and coordinated perioperative management are critical determinants of outcome.

Overall, our findings support the view that SPE remains an important therapeutic option for high-risk PE, particularly when delivered within specialised centres equipped with multidisciplinary pathways and the capability for rapid surgical intervention. As contemporary outcomes continue to improve, the role of SPE may shift from a salvage therapy toward a more proactive option in selected patients at high risk of deterioration.

Limitations

This study has several important limitations inherent to its retrospective, single-centre cohort design. The small sample size of 13 patients accrued over a 14-year period limits the representativeness and generalizability of the findings. In addition, the limited sample size and low number of outcome events result in reduced statistical power, precluding robust inferential analyses and limiting the ability to control for potential confounding factors; therefore, observed associations should be interpreted cautiously. The retrospective nature of the analysis introduces the risk of selection and information bias, as data collection relied on existing medical records that may contain incomplete or inconsistent documentation of clinical variables and follow-up outcomes. As a single-centre study conducted at a tertiary care institution, the findings reflect institution-specific practices, referral patterns, and surgical expertise, further limiting generalizability. Additionally, the highly selective referral of patients for surgical pulmonary embolectomy - often involving individuals with more severe presentations or failure of prior therapies - results in an intrinsically high-risk cohort, which may have influenced observed mortality and complication rates. Despite these limitations, this exploratory, descriptive analysis provides valuable insight into surgical pulmonary embolectomy in a specialized setting.

## Conclusions

This 14-year single-centre experience supports the continued role of surgical pulmonary embolectomy as an important treatment for selected patients with acute high-risk pulmonary embolism. Despite the severity of illness in the cohort, outcomes - including an in-hospital mortality of 23.1% and 12-month survival of 76.9% - were comparable to those reported by other specialized centres. These findings highlight the value of timely surgical intervention and coordinated multidisciplinary care. Although limited by its small, retrospective, single-centre design, the study adds to the evidence supporting SPE, particularly when thrombolytic or catheter-based therapies are unsuitable or unsuccessful. Nonetheless, in view of the critical nature of surgical pulmonary embolectomy and the exploratory, descriptive nature of this retrospective single-centre study, the findings should be interpreted with caution.

## References

[1] Goldberg JB, Giri J, Kobayashi T (2023). Surgical management and mechanical circulatory support in high-risk pulmonary embolisms: historical context, current status, and future directions: a scientific statement from the American Heart Association. Circulation.

[2] Fukuda I, Daitoku K (2017). Surgical embolectomy for acute pulmonary thromboembolism. Ann Vasc Dis.

[3] Lumsden AB, Suarez E (2016). Interventional therapy for pulmonary embolism. Methodist Debakey Cardiovasc J.

[4] Sharp EH (1962). Pulmonary embolectomy: successful removal of a massive pulmonary embolus with the support of cardiopulmonary bypass—case report. Ann Surg.

[5] Götzinger F, Lauder L, Sharp AS (2023). Interventional therapies for pulmonary embolism. Nat Rev Cardiol.

[6] Konstantinides SV, Meyer G, Becattini C (2020). 2019 ESC guidelines for the diagnosis and management of acute pulmonary embolism developed in collaboration with the European Respiratory Society (ERS). Eur Heart J.

[7] Branigan B, Brown S, Zavala R, Merritt H (2023). Contemporary management of intracardiac thrombi: a tale of two clots. J Extra Corpor Technol.

[8] Giri J, Sista AK, Weinberg I (2019). Interventional therapies for acute pulmonary embolism: current status and principles for the development of novel evidence: a scientific statement from the American Heart Association. Circulation.

[9] Rahouma M, Al-Thani S, Salem H (2024). The outcomes of surgical pulmonary embolectomy for pulmonary embolism: a meta-analysis. J Clin Med.

[10] Panholzer B, Gravert H, Borzikowsky C (2022). Outcome after surgical embolectomy for acute pulmonary embolism. J Cardiovasc Med (Hagerstown).

[11] Motekallemi A, Rohrwild LC, Ajouri J (2025). Surgical pulmonary embolectomy versus systemic thrombolysis in high-risk pulmonary embolism: a retrospective single-center analysis. J Clin Med.

[12] Ishida K, Nishimoto Y, Ohbe H (2025). Short-term outcomes of thrombolysis versus surgical pulmonary embolectomy in patients with high-risk pulmonary embolism. J Thorac Cardiovasc Surg.

[13] Pollack CV, Schreiber D, Goldhaber SZ (2011). Clinical characteristics, management, and outcomes of patients diagnosed with acute pulmonary embolism in the emergency department: initial report of EMPEROR (multicenter emergency medicine pulmonary embolism in the real world registry). J Am Coll Cardiol.

[14] Harjola VP, Mebazaa A, Čelutkienė J (2016). Contemporary management of acute right ventricular failure: a statement from the Heart Failure Association and the working group on pulmonary circulation and right ventricular function of the European Society of Cardiology. Eur J Heart Fail.

[15] Anderson FA Jr, Spencer FA (2003). Risk factors for venous thromboembolism. Circulation.

[16] Heit JA, Spencer FA, White RH (2016). The epidemiology of venous thromboembolism. J Thromb Thrombolysis.

[17] White RH (2003). The epidemiology of venous thromboembolism. Circulation.

[18] Tak T, Karturi S, Sharma U, Eckstein L, Poterucha JT, Sandoval Y (2019). Acute pulmonary embolism: contemporary approach to diagnosis, risk-stratification, and management. Int J Angiol.

[19] Argyriou A, Vohra H, Chan J, Ahmed EM, Rajakaruna C, Angelini GD, Fudulu DP (2024). Incidence and outcomes of surgical pulmonary embolectomy in the UK. Br J Surg.

[20] Akhlaghpasand M, Mohammadi I, Hajnorouzali A (2025). Salvage pulmonary embolectomy following cardiac arrest: a 10-year experience. Ann Med Surg (Lond).

[21] Bloomfield P, Boon NA, de Bono DP (1988). Indications for pulmonary embolectomy. Lancet.

[22] Torbicki A, Galié N, Covezzoli A, Rossi E, De Rosa M, Goldhaber SZ (2003). Right heart thrombi in pulmonary embolism: results from the international cooperative pulmonary embolism registry. J Am Coll Cardiol.

[23] Konstantinides S, Geibel A, Kasper W, Olschewski M, Blümel L, Just H (1998). Patent foramen ovale is an important predictor of adverse outcome in patients with major pulmonary embolism. Circulation.

[24] Chatterjee S, Chakraborty A, Weinberg I (2014). Thrombolysis for pulmonary embolism and risk of all-cause mortality, major bleeding, and intracranial hemorrhage: a meta-analysis. JAMA.

[25] Jimenez D, Bikdeli B, Marshall PS, Tapson V (2018). Aggressive treatment of intermediate-risk patients with acute symptomatic pulmonary embolism. Clin Chest Med.

[26] Tan JS, Liu N, Hu S (2022). Association between the use of pre- and post-thrombolysis anticoagulation with all-cause mortality and major bleeding in patients with pulmonary embolism. Front Cardiovasc Med.

[27] Kadner A, Schmidli J, Schönhoff F, Krähenbühl E, Immer F, Carrel T, Eckstein F (2008). Excellent outcome after surgical treatment of massive pulmonary embolism in critically ill patients. J Thorac Cardiovasc Surg.

[28] Vohra HA, Whistance RN, Mattam K (2010). Early and late clinical outcomes of pulmonary embolectomy for acute massive pulmonary embolism. Ann Thorac Surg.

[29] Meneveau N, Séronde MF, Blonde MC (2006). Management of unsuccessful thrombolysis in acute massive pulmonary embolism. Chest.

[30] Takahashi H, Okada K, Matsumori M, Kano H, Kitagawa A, Okita Y (2012). Aggressive surgical treatment of acute pulmonary embolism with circulatory collapse. Ann Thorac Surg.

